# Machinability Investigations of Inconel-800 Super Alloy under Sustainable Cooling Conditions

**DOI:** 10.3390/ma11112088

**Published:** 2018-10-25

**Authors:** Munish Kumar Gupta, Catalin Iulian Pruncu, Mozammel Mia, Gurraj Singh, Sunpreet Singh, Chander Prakash, P. K. Sood, Harjot Singh Gill

**Affiliations:** 1 Mechanical Engineering, NIT Hamirpur, Hamirpur 177005, India; munishguptanit@gmail.com or munishguptae7876@cumail.in (M.K.G.); munishbb@gmail.com (P.K.S.); 2 Mechanical Engineering, Chandigarh University, Gharuan 140413, Punjab, India; harjot.gill@cumail.in; 3 Mechanical Engineering, Imperial College London, Exhibition Rd., SW7 2AZ London, UK; 4 Mechanical Engineering, School of Engineering, University of Birmingham, B15 2TT Birmingham, UK; 5 Mechanical & Production Engineering, Ahsanullah University of Science and Technology, Dhaka 1208, Bangladesh; mozammelmiaipe@gmail.com; 6 School of Mechanical Engineering, Lovely Professional University, Phagwara 144411, Punjab, India; singh_gurraj@yahoo.co.in (G.S.); snprt.singh@gmail.com (S.S.); chander.mechengg@gmail.com (C.P.)

**Keywords:** machining, cutting force, tool wear, surface roughness, chip control

## Abstract

With regard to the manufacturing of innovative hard-machining super alloys (i.e., Inconel-800), a potential alternative for improving the process is using a novel cutting fluid approach. Generally, the cutting fluids allow the maintenance of a better tool topography that can generate a superior surface quality of machined material. However, the chemical components of fluids involved in that process may produce harmful effects on human health and can trigger environmental concerns. By decreasing the cutting fluids amount while using sustainable methods (i.e., dry), Near Dry Machining (NDM) will be possible in order to resolve these problems. This paper discusses the features of two innovative techniques for machining an Inconel-800 superalloy by plain turning while considering some critical parameters such as the cutting force, surface characteristics (*R_a_*), the tool wear rate, and chip morphology. The research findings highlight the near-dry machining process robustness over the dry machining routine while its great potential to resolve the heat transfer concerns in this manufacturing method was demonstrated. The results confirm other benefits of these methods (i.e., NDM) linked to the sustainability aspects in terms of the clean process, friendly environment, and permits as well as in terms of improving the manufacturing characteristics.

## 1. Introduction

The Inconel-800 alloy that is part of a large group of nickel-based super alloys is identified in some specialized aeronautical applications especially on the turbine and aero-components. This material is employed in this field due to its excellent properties such as good resistance to corrosion and high chemical as well as physical strength against elevated temperatures [1]. However, some inherent characteristics such as very high toughness combined with a reduced heat transfer coefficient and limited work hardening put this alloy in the category of high susceptibility and is not to be considered for machinability [2,3,4,5]. In other words, the key aim of super alloys machining is to achieve high efficiency, good surface finish, and an economical manufacturing cost [6,7,8,9]. Between numerous machining parameters with great influence on the previously mentioned objectives, the approach of cutting the fluid emerges out as the suitable alternative [10,11,12,13,14,15]. In the machining process, the cutting fluids permit keeping tool life integrity longer and, thus, generates better surface quality [16,17]. However, some concerns were risen because of potential adverse effects in the human environment presence because the cutting fluids can contain potentially harmful chemical ingredients [18]. A survey released recently on the aerospace sector, which considers the manufacturing process, reveals an increase in the annual expenditure on the cutting fluids up to several percentages (i.e., 20%), which highlights the importance of the overall cutting tool process and lubrication [17,19]. Therefore, in order to obtain a green, sustainable, and clean environment, it may be necessary to use dry machining and/or near dry machining that is quite often regarded as a potential alternative effective strategy [20,21,22]. 

So-called, dry machining generates the manufacturing process while the cutting fluids may completely be eliminated. Yet, this process can generate high temperatures during the machining process and when producing leads to greater concern about the machining of nickel super alloys (i.e., limited tool life combined with low surface quality) [23]. Hence, the application of the advanced process (i.e., near dry machining (NDM) and/or the minimum quantity lubrication (MQL)) will be possible to tackle the process limitations when it is used for dry machining. When machining conventional alloys, NDM has shown promising results [24,25]. In the NDM technique, the lubrication regime is produced by applying the mist form of the cutting oil particles in the cutting zone (tool-work interface) while using the compressed gas. The limited quantity of the coolant generates less waste compounds as well as several other advantages (i.e., ecological and potentially biological benefits). In addition, a smaller quantity of coolant permits us to save money that can be used for the chip recycling purpose [3]. Several researchers including Li et al. [26], Devillez et al. [27], Thakur et al. [28], Zhang et al. [29], Pusavec et al. [30], Tazehkandi et al. [31], Tazehkandi et al. [13], Pusavec et al. [32], and Marques et al. [33] have performed various machining experiments on Inconel alloys by considering dry and MQL conditions.

Table 1 clearly manifests that the application of sustainable methods (dry and near dry machining) is mostly performed on various grades of Inconel alloys such as Inconel-718, Inconel-706, Inconel-738, Inconel-825, and Inconel-725. The literature review confirms the limited amount of research dedicated to the Inconel-800 superalloy and no evidence for manufacturing of this alloy by the turning process. However, there was emphasis regarding the importance of using an Inconel-800 superalloy in the manufacturing of turbine blades and numerous aerospace parts. Therefore, this survey proposes investigating the importance of machining characteristics (i.e., cutting forces, surface morphology and its roughness, details of chip control, and the rate of tool wear) when the Inconel-800 superalloy is machined by considering two sustainable methods i.e., dry and near-dry machining.

## 2. Materials and Methods 

### 2.1. Details of Cutting Inserts and the Workpiece Materials 

**Figure 1:**
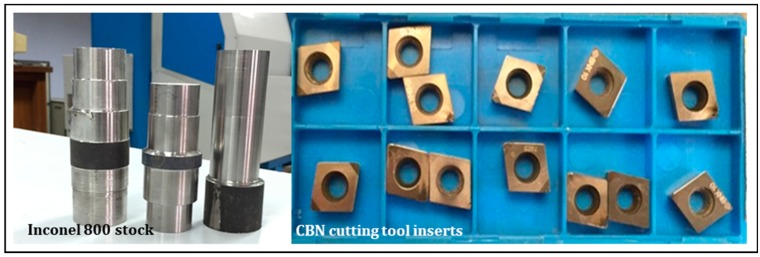
Inconel-800 super alloy work material and CBN inserts.

The Inconel-800 superalloy made as a bar with its nominal dimension of 50 mm diameter and a length of 150 mm length was employed in this research. Figure 1 presents details of the machined material profile. The hardness of this material is around 42 HRC. Table 2 indicates the chemical composition of the studied material. Further details concerning the heat treatment conditions of Inconel-800 are presented in Table 3, respectively. The inserts configuration made of cubic boron nitride are bounded by the following details: a positive angle of 7°, the nose radius of 0.4 mm, its clearance is around 80°, having a rhombic shape, and denoted CCGW 09T304-2 were engaged in the experiment due to its superior characteristics when compared to other inserts (see its configuration as per Figure 1).

### 2.2. NDM Features and the Turning Procedure

**Figure 2:**
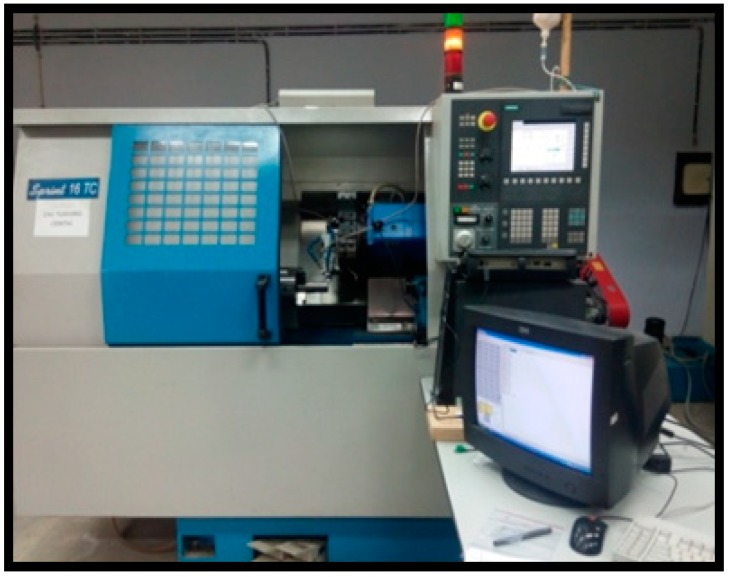
Experimental setup [35].

**Figure 3:**
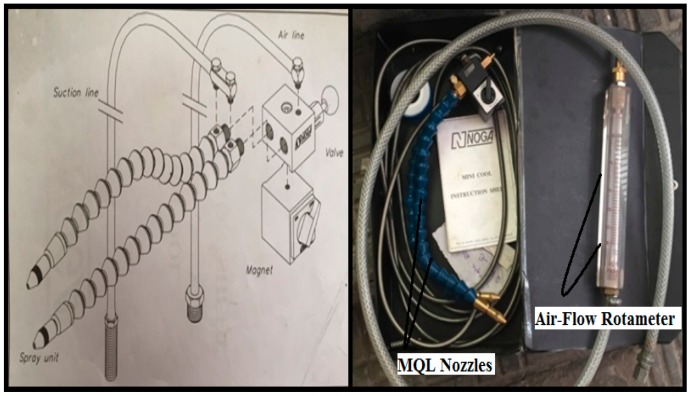
Schematic diagram of NDM (i.e.,MQL)nozzles with air flow rota-meter [18].

Figure 2 presented the entire turning machine under which the machining of the superalloy was investigated. This is a high precision CNC center lathe machine identified as Sprint 16TC, Batliboi, Mumbai, India) that is equipped with a special Siemens control system. The manipulation of the tool is realized by three simultaneously axes (*X*, *Y* and *Z* axis).The NDM (i.e., MQL) apparatus is a professional NOGA NDM system (as exhibited in Figure 3) that is working by using a cutting oil as water soluble (i.e., Balmerol, Kolkata, India) and has a working ratio of 20:1. In this machining routine, the procedure might incorporate a 300 mL/h flow rate of cutting fluid, an air flow average of about 60 L/min, and a pressure of 5 bar that were settled for all turning experiments. 

### 2.3. The Control of the Main Parameters 

A TeLC DKM2010 dynamometer (Unna, Germany) connected to XKM software was used to measure the main cutting force (*F_c_*). The morphology of the main tool flank wear (*V_bmax_*) was detected by a microscope (Mitutoyo, Kawasaki, Japan) while details of surface roughness (*R_a_*) value measurements were made by using a surface roughness tester (Mitutoyo SJ 301, Kawasaki, Japan) for a cut-off length of 0.8 mm. For a consistent evaluation, the finished work piece was as a set on three different points with respect to the tool displacement while a mean calculation was later implemented.

### 2.4. Process Parameters Selection

Two main factors were selected such as the cutting speed and feed rate, which may generate valuable responses in terms of machining conditions. Three different fixed cutting speeds were employed since 200 m/min considered low speed, a medium one of 250 m/min, and one higher of 300 m/min. These ranges of the above speeds were selected because an early tool failure occurred when the speed limit is more than 300 m/min. By contrast, when the speed is lower than 200 m/min, no early failure occurs. However, it is not practical for the industry. Yet, other parameters (i.e., the feed rate) were selected in conformity with the literature statements that vary as 0.10, 0.15, and 0.20 mm/rev. The depth of the cut was settled to fix 1 mm in all the experiments.

## 3. Results

### 3.1. Dry and Near Dry Machining Effect on the Main Cutting Force

**Figure 4:**
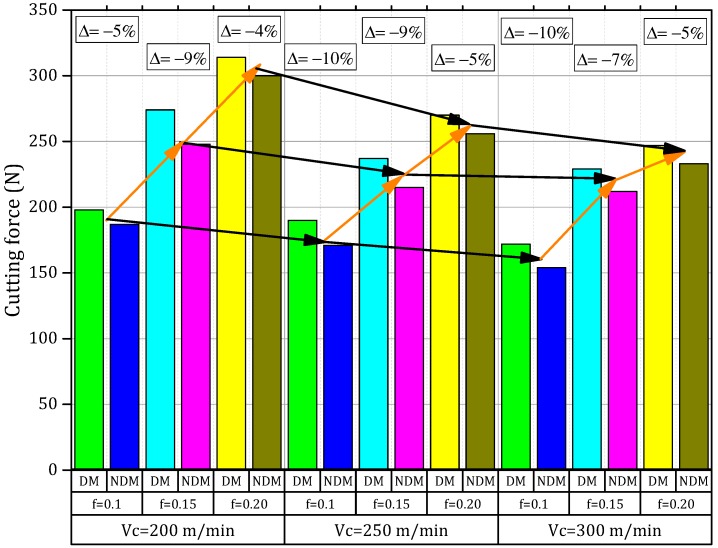
Effects of change of cutting speed, feed rate, and cooling/lubrication conditions on the cutting force.

The main problems in manufacturing a dedicated machine tool apparatus may arise as the cause of cutting forces responsible for different deformation, which can affect the work-piece and are estimated by tolerance violations and errors. Their major impact is in a close relationship with some material structural parameters (i.e., work-piece material properties, tool material geometry and the material itself, cutting fluid properties, and strategy of machining) and/or conditions of cutting. The values of the cutting force obtained in dry and near dry conditions by using alternative cutting parameters were introduced in Figure 4. During machining, the Inconel-800 superalloy was observed as a reduction in the cutting forces values when the cutting speed is high while the feed rates decrease. Thereby, it generates a reduction of the coefficient of friction at the cutting zone because of the quick motion that occurs on the work-piece. This routine requires only a minimum amount of cutting force to produce the shear in the work-piece [36]. Furthermore, a growth in the feed rate raises the main cutting force because the tool-chip contact length is extended by an increased feed rate. Hence, high cutting forces are required to plough a large section of the chips. This ploughing procedure results in higher stresses and deformation of the layer being cut, which causes a high cutting force. Moreover, the near dry machining process generates low cutting force values i.e., 4% to 9% when compared with dry machining conditions (see details in Figure 4).

The difficulty of machining the Inconel-800 alloy is demonstrated as well as by an excess of heat produced during dry machining that can severely change the work-piece into a more brittle material, which definitely raises the cutting forces [31]. However, by using the near dry machining strategy including a cutting fluid and compressed air, it is possible to reduce significantly the temperature in the core of the cutting zone, which maintains the integrity of tool material (in terms of hardness). This includes decreasing the potential adhesion between interacting surfaces. The similar results were reported by Gupta et al., i.e., overall reduction of cutting forces of 5% to 15% in machining of a titanium grade (2) alloy [3,18]. In addition, the mist form of cutting fluid permits to generate a jet having a high-speed through its nozzles, which will help to reinforce the capillary of the tool-chip interface. Therefore, this allows the reduction of friction values and cutting forces by forming the boundary-lubricating layer. This improvement is in good agreement with the results presented by Tazehkandi et al., [37,38] in machining of the Inconel-725 and 740 alloy.

### 3.2. Surface Roughness Generated from Dry and Near Dry Machining

**Figure 5:**
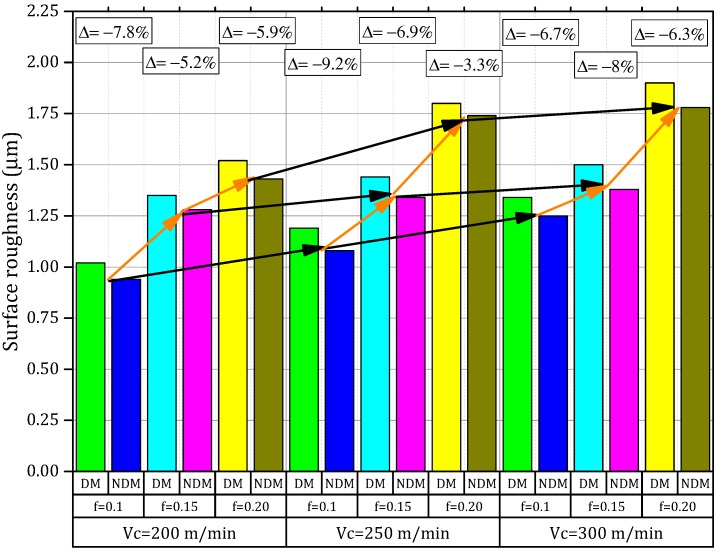
Effects of the change of cutting speed, the feed rate, and the cooling/lubrication conditions on surface roughness.

**Figure 6:**
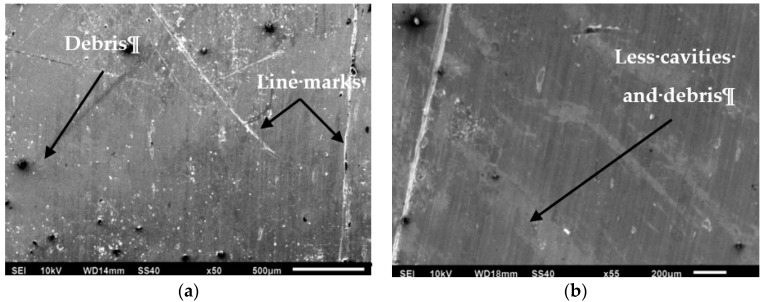
SEM micrographs of machined surfaces at *V_c_*= 300 m/min, *f* = 0.2 mm/rev: (**a**) dry; (**b**) MQL.

By using the ISO 4287 standard guidance, it was possible to estimate the average of the surface roughness (*R_a_*) [16]. Figure 5 shows the values of surface characteristics (roughness *R_a_*) evaluated when it was employed with a dry and near dry cooling/lubricated strategy. Furthermore, morphological details of machined surfaces obtained by SEM microscopy (Jeol, Tokyo, Japan) and produced for different conditions such as dry and NDM are introduced in Figure 6. The details from images from Figure 6 demonstrated the effects of cooling conditions on the end surface finish. Furthermore, when the cutting speed was raised along with an increase in the feed rate, higher surface roughness values were generated.

This fact (i.e., the issue when the rise is in the cutting speed and feed rate) causes the potential vibration at the machine tool assembly and the rubbing between the tool and work-piece may produce a local increase of the temperature on the cutting zone that later on will generate loss of surface integrity of the work-piece. Dry and near dry machining outcomes in terms of surface roughness (from Figure 5 and Figure 6) prove the benefits of the surface quality when an Inconel-800 is machined under NDM conditions (i.e., reduction of values from 3% to 10%) that are far better than those of dry machining. The performances were validated by Gupta et al. [18,35] in turning of titanium (grade-2) alloy under MQL conditions.

The NDM process generated smaller values of surface roughness with no cavities and metal debris at all cutting conditions. In fact, when the pressurized air is introduced together with cutting fluid, it generates a significantly better cooling condition on the cutting zone. On both works, the materials were emphasized with a small thermal softening that has decreased to a certain extent by reducing the friction between the tool and the work-piece interface. Hence, a high surface finish is observed in NDM conditions. It can also be noted that the variation is more obvious under dry conditions. The means, the higher tensile strength (465 MPa), and the hardness (42 HRC) of the Inconel-800 can produce some undesirable effects on the surface quality by increasing the built-up-edge (BUE) tendency when considering dry conditions. Nevertheless, this phenomenon was reduced by applying near dry machining that allows as well as diminishs the tendency of the BUE formation and generates better surface quality [26]. The results reported by the Mia et al. [12] in machining of hardened steel confirms this enhancement mechanism.

### 3.3. Tool Wear Evaluation on Dry and Near Dry Machining

**Figure 7:**
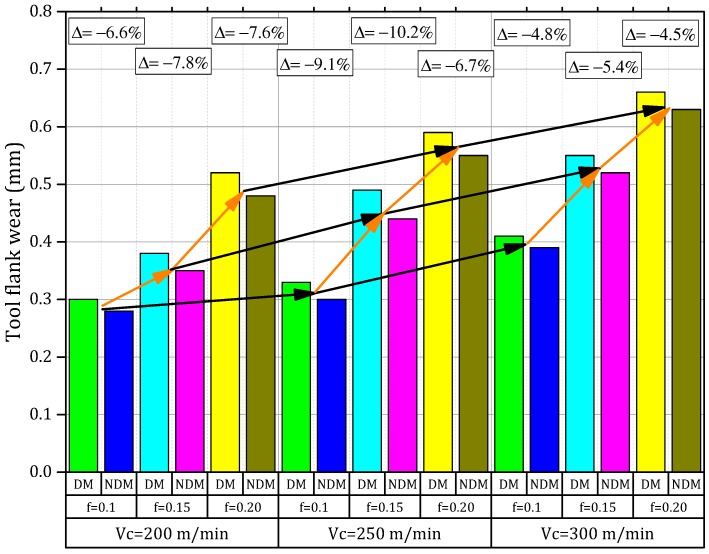
Effects of change of cutting speed, feed rate, and cooling/lubrication conditions on tool flank wear.

Mechanistically and physicality demonstrated that the machining process cannot occur without generating a certain amount of heat. The hard contact between the tool-chip interfaces can produce the maximum heat. The heat produced there can affect directly the integrity of tool life, the variation of cutting forces, and/or the geometry and its morphology of the chip. It was demonstrated that the main type of tool failure is of adhesion, abrasion, or diffusion. The value of tool wear with variable cutting speeds at different feed rates is demonstrated in Figure 7. 

**Figure 8:**
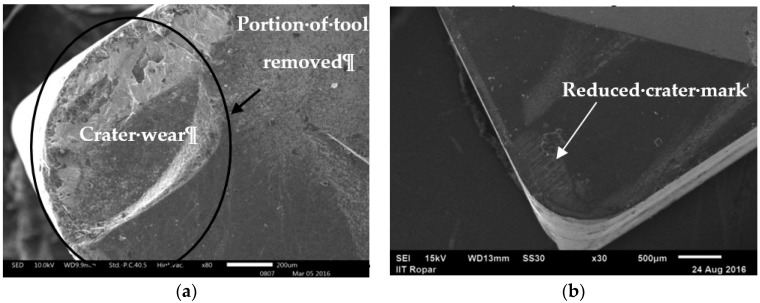
SEM of tool wear while machining Inconel-800 alloy at *V_c_*= 300 m/min, *f* = 0.2 mm/rev: (**a**) dry; (**b**) MQL.

It has been observed from the plot that the tool wears rapidly and grows with the rise in cutting speed combined with the growth of the feed rate. The reasons might be linked to the fact that, when the cutting speed together with the feed rate was increased, an extension of the chip area occurred while the friction between tool-chip interfaces increased. Therefore, *V_bmax_* grew. In addition, it was observed that partial de-bonding of the protecting coating that covers the tool and is caused by the high speed–feed combination may further increase the amount of flank wear. The comparisons of tool wear considering dry and near dry conditions in Figure 8 exhibited that the tool wear of Inconel-800 under NDM conditions is lower than i.e., a reduction of 4% to 11% than those of dry machining. 

The higher hardness values of the Inconel-800 (42 HRC) can be detrimental to the tool life of the dry machining, which reduces the strain rate of the tool such as, when it reaches a certain limit, the damage will be clearly described by the accentuated tool wear. The mechanism observed is proven by Wang et al. [39] in the machining of the Inconel-182 alloy. The NDM approach produces a better working condition due to the mist of lubricant by a proper penetration of compressed air that occurs at the tool-chip interface. This later mechanism works as a suitable tool for the cooling strategy by generating a better lubrication. Once the temperature in the cutting zone is reduced, it is possible to decrease the stickiness level on the work-piece material. A low cutting temperature can maintain the structural integrity of tool materials (i.e., the hardness and its strength). By increasing the tool life, it allows a better process that presents a lower rate of susceptibility to an adhesion mechanism and most probably lower chance of bonding between the work piece materials to the surface of the tool that is more advantageous when compared with dry machining. Some deep craters were identified in the SEM analysis that corresponds to the rake faces and their cutting edge (as shown in Figure 8) as well as the nose, which almost broke away under dry conditions likely due to the high-temperature variation and an uncontrolled friction produced in the cutting zone. Whereas in near-dry machining, the use of compressed air with cutting fluid almost eliminates the craters’ patterns on the rake surface because of the most proficient diffusion of the coolant at the interface of the rake surface and the generated chip [26].

### 3.4. Chip Control on Dry and Near Dry Machining 

In metal cutting operations, the chip control is one of the most vital perspectives. The type of chip formed significantly affects the efficiency of the industrial machining process. The generation of chips that includes its aspect of breaking is prominent in machining since it can affect the tool life and the geometry of the surface finish that is linked to the accuracy of the work piece produced [32]. Hence, the formation of a suitable type of chips with good chip control is crucial in metal cutting operations. By machining, the Inconel-800 with the NDM approach was possible by crushing the chips into very small fragments that have an average bulk ratio of 8.1 while some unbroken, long continuous chips that have a bulk ratio of circa. 68 were produced under machining dry conditions, which is presented in Table 4. 

**Figure 9:**
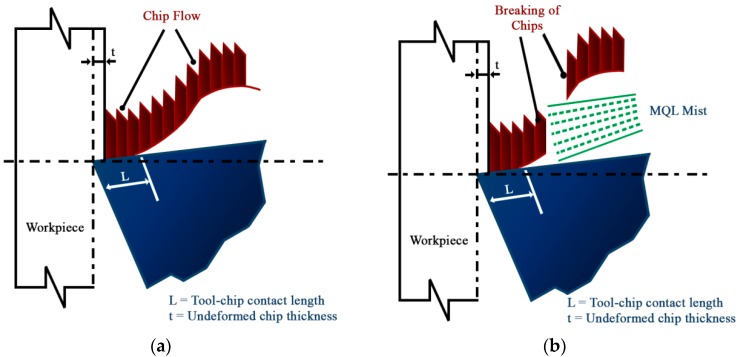
Schematic diagram of the chip formation under (**a**) dry and (**b**) MQL conditions [3].

Figure 9 provides insight for chip formation for the machining process confirming that no static pressure was applied when it was machined under dry conditions. The chip breakage occurs in the dry condition likely because of the hard contact between the chips with an obstruction leading to a shear plane fracture due to a negative bending moment produced that results in a high bulk ratio of chips. However, in the case of near-dry machining, the high-pressure developed by the jet mixture will be enough to start fragmentation of the chip and will create a positive bending moment that may occur before to making a hard contact with an obstruction. It seems that the NDM approach generates a suitable form of chips due to high breakability, i.e., with geometry that represents a small helical chip type. The dry conditions of machining produce the chips with a blue color that may be because of the high-temperature that occurs in the deformation zone. However, NDM conditions generate the chips with a light golden color that is due to the large reduction of a tool-chip interface temperature. The overall benefits of NDM approach are linked to a large reduction in the adhesion mechanism, a better-cushioning effect, and a constant friction level that leads to a lower built-up edge formation that is depicted in Figure 8.

## 4. Conclusions

Near-dry machining or NDM represent a sustainable strategy that could reduce several machining issues related to its performance i.e., cutting forces, surface roughness, tool wear etc. The main findings of the present investigations are summarized below.

(1) For the cutting forces, an application of the NDM produced less magnitude of cutting forces (i.e., reduction in 4% to 9%) than those of the dry machining environment. The cutting fluids that are of mist form a pass with a high-speed jet through the nozzles, which will help to reinforce the lubrication at the capillary interface between the tool and chip, which reduces the friction and cutting forces by forming the boundary lubricating layer.
(2) In NDM conditions, the machined surface shows a greater quality (i.e., reduction of surface roughness from 3% to 10%) because a proper usage of the cutting fluid within a very limited quantity i.e., near dry machining can possibly enhance the surface quality by reducing the gradient temperature in the cutting area and avoids early damage of the tool-tip. Moreover, an enhanced lubrication due to the oil droplets presumably reduced the friction between the fraying surfaces.
(3) After analyzing the tool wear, the NDM reveals better performances in terms of the tool wear value (reduction of 4% to 11%) in contrast with dry machining. The use of compressed air with cutting fluid has almost eliminated the amount of the crater on the rake surface by using a proficient dispersion of the coolant between the rake surface and the chip formed.
(4) For the analysis of chip control, the application of near-dry machining generates small fragments of chips with a bulk average ratio of 8.1 while some unbroken, very long continuous chips that have a bulk ratio of approximately 68 were produced under machining dry conditions.
(5) The near dry machining process generates dry chips that can be considered as an initiative towards the cleaner production.

## Figures and Tables

**Table 1:** Literature survey/work done with respect to machining various grades of Inconel super alloys.

Author and Year	W/P & Tool Material	Cutting Parameters	Cooling Types	Work Done/Investigations	Results/Remarks
Li et al., 2006 [26]	Inconel-718, coated carbide inserts	Milling	Dry	Tool wear & cutting force	-
Devillez et al., 2011 [27]	Inconel-718, coated carbide inserts	*V_c_*: 40, 60, 80 m/min, *f*: 0.1 mm/rev, *a_p_*: 0.5 mm	Dry and wet	Cutting forces, surface roughness, Surface quality, Tool life Residual stresses etc.	Dry machining with a coated carbide tool leads to potentially acceptable results
Thakur et al., 2015 [28]	Inconel 825, Coated and uncoated carbide inserts	*V_c_*: 51, 84, 124 m/min, *f*: 0.198 mm/rev; *a_p_*: 1 mm	Dry	Chip morphology, chip thickness ratio, tool wear, surface, and sub-surface integrity	Coated tools were superior when compared with uncoated tools
Zhang et al., 2012 [29]	Inconel-718, Coated Carbide Inserts	Milling operation: *V_c_*: 55 m/min, feed/tooth:0.1 mm/tooth, *a_p_*: 0.5 mm(axial), 1 mm (radial)	Dry, MQL	Tool life and cutting force	Improved tool life with lower cutting forces due to the MQL conditions
Pusavec et al., 2014 [30]	Inconel-718, Coated Carbide Inserts	*V_c_*: 30, 60, 90, 120 m/min, *f*: 0.05, 0.12, 0.18, 0.25 mm/rev, *a_p_*: 0.2, 0.63, 1.07, 1.50 mm	Dry, MQL, Cryo, Cryo-lubrication	Tool-wear, surface roughness, cutting forces, chip breakability measurements	Cryo-lubrication shows the most beneficial performances because of low temperature of liquid nitrogen
Tazehkandi et al., 2015 [31]	Inconel-706, Coated Carbide Inserts	*V_c_*: 30, 50, 70, 90 m/min, *f*: 0.08, 0.10, 0.12, 0.14 mm/rev, *a_p_*: 0.1, 0.4, 0.7 mm	Wet, MQL	Cutting force, surface roughness, and cutting temperature	By using MQL, the values of selected responses are lower than wet cooling
Tazehkandi et al., 2015 [34]	Inconel-783, PCBN Inserts	*V_c_*: 60, 100, 140, 180 m/min, *f*: 0.1, 0.15, 0.20, 0.25 mm/rev, *a_p_*: 0.2, 0.6, 1, 1.4 mm	Wet, MQL	Cutting force, surface roughness, and cutting temperature	Utilizing a PCBN tool in MQL model can reduce the selected responses
Pusavec et al., 2015 [32]	Inconel-718, Coated Carbide Inserts	*V_c_*: 30, 60, 90, 120 m/min, *f*: 0.05, 0.12, 0.18, 0.25 mm/rev, *a_p_*: 0.2, 0.63, 1.07, 1.50 mm	Dry, MQL, Cryo, Cryo-lubrication	Tool-wear, surface roughness, cutting forces, chip breakability measurements, MRR	-
Marques et al., 2015 [33]	Inconel-718, Coated Carbide Inserts	*V_c_*: 100 m/min, *f*: 0.15 mm/rev, *a_p_*: 1.5 mm	Wet, MQL, MQSL	Tool-wear, surface roughness, cutting forces, and micro-hardness	MQSL shows very promising results followed by MQL and wet machining

**Table 2:** Chemical composition of Inconel-800 super alloy.

**Element**	Ni	Cr	Fe	C	Al	Ti	Al + Ti
**Composition (%)**	30.0–35.0	19.0–23.0	39.5 min	0.10 max	0.15–0.60	0.15–0.60	0.30–1.20

**Table 3:** Heat treatment conditions of Inconel-800 super alloy.

Heat Treatment	Intermediate Treatment	Final Treatment
1050 °C for 2 h, air cool	850 °C for 6 h, air cool	700 °C for 2 h, air cool

**Table 4:** Chip shapes obtained under dry and MQL conditions.

Cutting Conditions	Dry	NDM
*V_c_*= 200 m/min *f* = 0.10 mm/rev	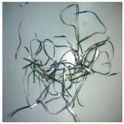	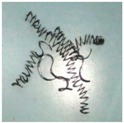
*V_c_*= 250 m/min *f* = 0.15 mm/rev	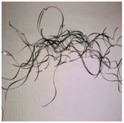	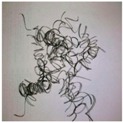
*V_c_*= 300 m/min *f* = 0.2 mm/rev	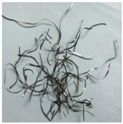	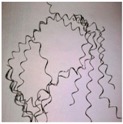
	Long continuous chips of a blue color	Small helical chips of a light golden color
	Average bulk ratio of 68	Average bulk ratio of 8.1
